# Investigating the Influence of Varying Surface Conditions on Human Postural Control and Sensory Integration Strategies

**DOI:** 10.3390/bioengineering11060618

**Published:** 2024-06-18

**Authors:** Seo-Yoon Park, Sang-Seok Yeo, Tae-Woo Kang, Dong-Kyun Koo

**Affiliations:** 1 Department of Physical Therapy, College of Health and Welfare, Woosuk University, 443 Samnye-ro, Samnye-eup, Wanju-gun 55338, Republic of Korea; pgy0614@hanmail.net (S.-Y.P.); ktwkd@hanmail.net (T.-W.K.); 2 Department of Physical Therapy, College of Health and Welfare Sciences, Dankook University, Cheonan 31116, Republic of Korea; yeopt@hanmail.net; 3 University-Industrial Cooperation Corps of HiVE Center, Wonkwang Health Science University, 514, Iksan-daero, Iksan-si 54538, Republic of Korea

**Keywords:** balance, perturbation, environmental challenges, adaptive response

## Abstract

This study investigated the effects of different surface conditions on postural stability in response to unexpected perturbations. Thirty healthy adults underwent balance assessments on flat, incline ramp, balance pad, and balance pad on incline ramp surfaces. The center of pressure (COP) displacement in the mediolateral (ML) and anteroposterior (AP) directions, the velocity, and the area were measured. We found that the flat and ramp conditions resulted in significantly lower COP ML (F(3, 87) = 38.272, *p* < 0.001, ηp^2^ = 0.569) and AP displacements (F(3, 87) = 89.177, *p* < 0.001, ηp^2^ = 0.755), velocity (F(3, 87) = 89.177, *p* < 0.001, ηp^2^ = 0.755), and area (F(3, 87) = 52.659, *p* < 0.001, ηp^2^ = 0.645) compared to the balance pad and balance pad on ramp conditions (*p* < 0.05). The use of a balance pad, particularly on a ramp, significantly increased all the COP measurements, suggesting greater challenges to postural control. Through these findings, we demonstrate the adaptability and limitations of the human postural control system in response to varying surface conditions and perturbations.

## 1. Introduction

Maintaining balance, which is crucial for safely performing daily activities, requires sophisticated coordination of the body’s center of gravity over its base of support [1]. This coordination is achieved through the integration of sensory inputs from the visual, vestibular, and proprioceptive systems, each of which plays a crucial role in detecting changes in body position and initiating corrective actions to preserve balance [2,3]. The interaction of sensory, motor, and nervous systems enables individuals to navigate environmental challenges and respond to disturbances, underscoring the importance of balance for human mobility and safety [4]. Effective balance control requires rapid postural adjustments in real-life scenarios where external perturbations, such as slips or trips, occur [5]. Visual information is crucial for anticipating disturbances, while the vestibular system monitors head movements, and proprioception provides detailed feedback on the body’s position; together, they facilitate precise balance control [6].

Recent studies have explored the impact of surface conditions on balance, revealing that unstable and inclined surfaces pose significant challenges [7,8]. The findings suggest a need for a deeper understanding of the impact of combined sensory disruptions on postural stability, especially when unexpected external perturbations occur on various surfaces [9]. However, current research has mainly concentrated on flat surfaces or the isolated effects of sensory impairments, resulting in a lack of knowledge regarding the integrated response to complex environmental conditions.

This study investigates the effects of different surface conditions on postural stability in the face of unexpected disturbances. We aim to improve the understanding of postural adjustment mechanisms across diverse environments by focusing on flat, inclined, and variously modified surfaces under visual occlusion. Current research has mainly concentrated on flat surfaces or the isolated effects of sensory impairments, resulting in a lack of knowledge regarding the integrated response to complex environmental conditions. We hypothesize that more challenging surfaces will lead to greater postural instability, and the absence of visual feedback will exacerbate the effects of surface conditions on balance control.

## 2. Materials and Methods

### 2.1. Participants

This study selected a total of 30 healthy adults in their 20s from a local university; the cohort consisted of 20 men and 10 women. The sample size was determined using G*Power (version 3.1.9.7) software with the following input parameters: effect size f = 0.25 (medium), α = 0.05, power = 0.80, and number of measurements = 4. The calculated sample size was 24 participants, but 30 participants were recruited to account for potential dropouts. The study included healthy adults in their 20s, with no specific age limit within this range. This age group was chosen to minimize the potential effects of age-related changes in postural control and to ensure a relatively homogeneous sample. The inclusion criteria for participants were: (1) no musculoskeletal injuries in the past 6 months, (2) no neurological damage in the past 6 months, (3) no vestibular disorders, and (4) no experiences of dizziness in the past 6 months. All participants were fully informed about the study’s objectives and procedures and provided informed consent in accordance with ethical principles. This study was approved by the Institutional Review Board of Dankook University (DKU 2023-03-024-005). The general characteristics of the 30 participants enrolled in this study are as presented in Table 1.

### 2.2. Balance Measurement

The study employed a professional balance assessment and training system (BT4-AP1173, Ab Hur Oy, Finland) to evaluate balance ability. The outcomes for measuring balance included the left–right displacement of the center of pressure (COP X) in millimeters (mm), the anterior–posterior displacement of the COP (COP Y) in mm, the trace length moved during a fixed measurement time divided by measurement time resulting in COP velocity (mm/s), and the area covered by the COP movement (C90 area) in square millimeters (mm^2^). Increases in these measurements indicate a decrease in postural and balance control capability, heightening the risk of falling.

### 2.3. Support Surfaces

Four types of surfaces were used to stimulate each sensory organ: (1) a force plate; (2) a 60 × 60 cm incline ramp at a 5° angle, which was placed on the force plate to create an inclined support surface; (3) a balance pad (Balance pad, Airex, Swiss) placed on the force plate to stimulate proprioceptive sensations; and (4) a balance pad on the incline ramp. All the experiments were conducted on the force plate.

### 2.4. Experimental Procedure

**Figure 1:**
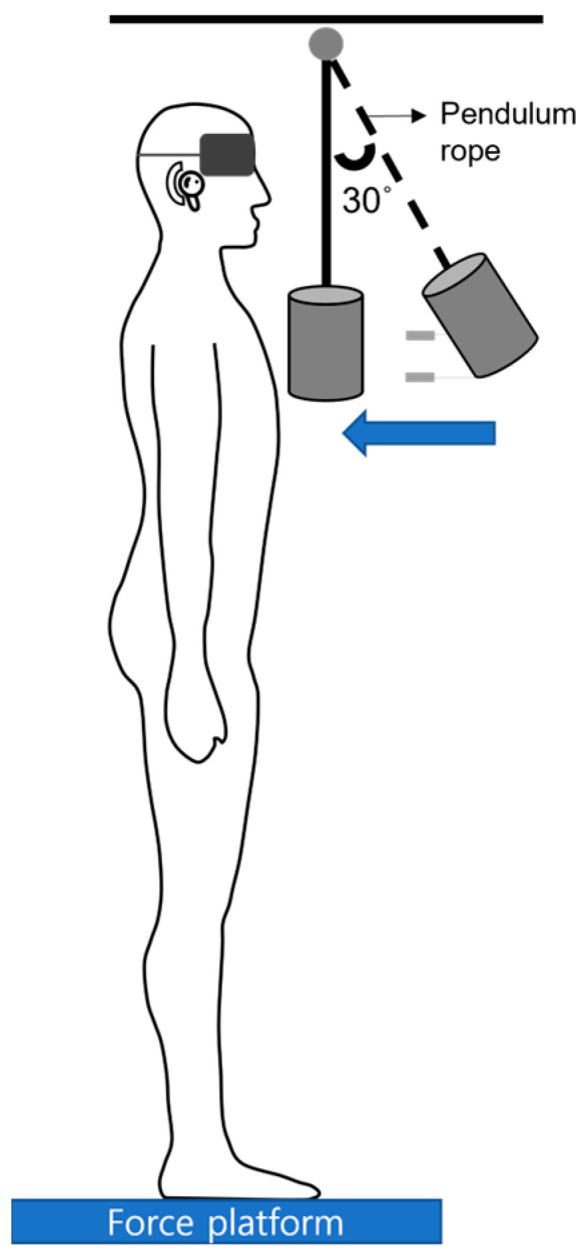
Schematic illustration of the experimental setup for inducing trunk perturbations using a pendulum impact system on participants standing on various support surfaces.

The experiment involved the participants standing barefoot on the force plate covered with four different surfaces, maintaining a natural foot distance while experiencing trunk perturbations. The perturbations were delivered using a pendulum attached to a cable suspended from the ceiling. The pendulum was positioned at a 30° angle from vertical, 0.6 m from the participant’s shoulder. A weight equivalent to 5% of the participant’s body mass was attached to the end of the pendulum and adjusted to hit the participant’s chest at shoulder level (Figure 1). For safety, a harness system loosely attached to the ceiling was used. A researcher released the pendulum following a “ready” signal, impacting the participant based on the pendulum’s motion principles, with a random 1–5 s delay to prevent predictability. The participants underwent familiarization with the pendulum perturbations twice before the experiment. Throughout the data collection sessions, the participants wore earphones to block environmental noise, which was primarily caused by the release of the pendulum, and blindfolds to block the view of the pendulum. The four support surfaces were applied randomly to the participants, and external perturbations were applied from the front to the back of the participants. The four support surfaces were applied randomly to the participants using a computer-generated sequence www.randomizer.org(accessed on 15 December 2023). Sealed envelopes containing the randomized sequences were prepared before the study and opened by a separate researcher at the start of each participant’s testing session. This procedure ensures transparency and prevents bias in the application of surface conditions. The researchers conducting the experiments and analyzing the data were blinded to the specific surface conditions being tested for each participant to further minimize potential bias. External perturbations were applied from the front to the back of the participants.

### 2.5. Data Analysis

The data collected in this study were analyzed using the IBM SPSS version 26.0 (IBM Corp., Armonk, NY, USA) statistical software. Descriptive statistics were used to present the general characteristics of the participants. The measures of normality were assessed using the Shapiro–Wilk statistic, with normality assumed at a value of *p* > 0.05. One-way repeated measures ANOVA was utilized to analyze the COP displacement in response to external perturbations on flat surfaces, incline ramps, balance pads, and balance pads on incline ramps, with Bonferroni correction for post hoc testing where significant differences were found. The significance level was set at 0.05.

## 3. Results

### 3.1. General Characteristics

The cohort consisted of 20 males (age: 24.70 ± 2.32 years, height: 176.00 ± 4.38 cm, body mass: 69.30 ± 8.47 kg) and 10 females (age: 23.50 ± 1.35 years, height: 162.60 ± 3.72 cm, body mass: 57.00 ± 9.18 kg).

### 3.2. COP Displacement

(1) Mediolateral COP X axis displacement

**Figure 2:**
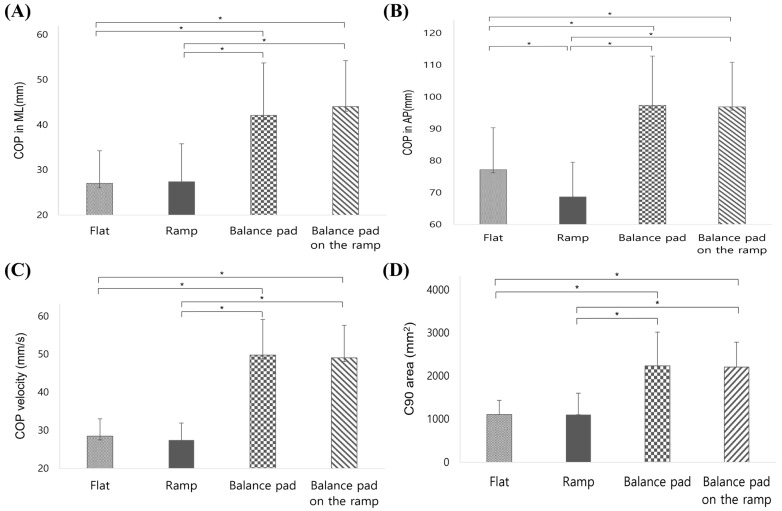
Results of the post hoc analysis comparing the COP displacement on different support surfaces (flat, incline ramp, balance pad, and balance pad on incline ramp) in response to external perturbations. (**A**) COP displacement in the ML (medio-lateral) direction. (**B**) COP displacement in the AP (antero-posterior) direction. (**C**) COP velocity in the ML direction. (**D**) COP area. Asterisks (*) indicate significant differences between support surface conditions (*p* < 0.05).

The comparison of the mediolateral displacement of the COP revealed statistically significant differences across the support surface conditions (F(3, 87) = 38.272, *p* < 0.001, ηp^2^ = 0.569). Post hoc analysis showed that the flat surface condition resulted in significantly lower displacement compared to the incline ramp, balance pad, and balance pad on incline ramp (*p* < 0.001). Additionally, the incline ramp condition exhibited significantly lower displacement than the balance pad (*p* < 0.001). Conversely, no significant difference was found between the incline ramp and balance pad on incline ramp conditions (*p* = 0.622) or between the balance pad and balance pad on incline ramp conditions (*p* = 0.698) (Figure 2A, Table 2).

(2) Anteroposterior COP Y axis displacement

The comparison of the anteroposterior displacement of the COP due to changes in the support surface showed statistically significant differences (F(3, 87) = 89.177, *p* < 0.001, ηp^2^ = 0.755). Post hoc analysis indicated that the flat surface condition had significantly lower displacement compared to the balance pad (*p* < 0.001). Both the flat and incline ramp conditions had significantly lower displacement than the balance pad and balance pad on incline ramp conditions (*p* < 0.001). There was no significant difference between the flat and incline ramp conditions (*p* = 0.065) (Figure 2B, Table 2).

(3) COP Velocity during Anteroposterior Perturbations

The comparison of COP velocity during anteroposterior perturbations across different support surfaces revealed statistically significant differences (F(3, 87) = 89.177, *p* < 0.001, ηp^2^ = 0.755). Post hoc analysis showed that the flat surface condition resulted in significantly lower COP velocity compared to the balance pad (*p* < 0.001). The incline ramp condition also resulted in significantly lower COP velocity compared to the balance pad and balance pad on incline ramp conditions (*p* < 0.001). There was no significant difference between the flat and incline ramp conditions (*p* = 0.938) or between the balance pad and balance pad on incline ramp conditions (*p* = 0.853) (Figure 2C, Table 2).

(4) C90 Area during Anteroposterior Perturbations

The comparison of the area of perturbation (C90 area) during anteroposterior perturbations across different support surfaces also showed statistically significant differences (F(3, 87) = 52.659, *p* < 0.001, ηp^2^ = 0.645). Post hoc analysis indicated that the flat surface condition had a significantly lower perturbation area compared to the balance pad (*p* < 0.001). The incline ramp condition also had a significantly lower perturbation area compared to the balance pad and balance pad on incline ramp conditions (*p* < 0.001). No significant difference was observed between the flat and incline ramp conditions (*p* = 0.834) or between the balance pad and balance pad on incline ramp conditions (*p* = 0.688) (Figure 2D, Table 2).

## 4. Discussion

This study aimed to investigate the effects of different surface conditions on postural stability in response to unexpected perturbations, to better understand the adaptability and limitations of the human postural control system in various environments. Our main findings reveal significant differences in COP displacement and velocity across surface types. Flat surfaces resulted in lower COP displacements and velocities compared to the incline ramp, balance pad, and balance pad on incline ramp conditions. These results provide valuable insights into the complex interplay between sensory feedback mechanisms and postural control strategies, highlighting the adaptability and limitations of the human postural control system in response to environmental challenges.

We observed that lower displacement on flat surfaces aligns with expectations, given the stability and predictability of these conditions, which likely minimizes the need for corrective postural adjustments [10]. This finding corroborates the theory that effective balance maintenance is facilitated by consistent sensory feedback, particularly from proprioceptive and vestibular inputs, which are less challenged on flat surfaces [11,12]. Conversely, the increased displacement on inclined and balance pad surfaces reflects the body’s adaptive response, which allows it to maintain equilibrium under less stable and more unpredictable conditions [13]. This adjustment likely involves heightened sensory processing and motor control efforts to counteract the destabilizing effects of the inclined and compliant surfaces [14]. The absence of significant differences between the incline and balance pad on incline conditions suggests a potential ceiling effect in the body’s capacity to compensate for combined instabilities, indicating that beyond a certain level of challenge, additional surface complexity does not proportionately increase mediolateral postural instability [12,15,16,17].

Moreover, the findings of lower displacement in flat and incline ramp conditions suggest that these environments provide a relatively more predictable basis for postural adjustment, even with the incline inducing a forward-leaning posture to maintain stability [18,19]. In contrast, the higher displacement observed on balance pads indicates the substantial impact of unstable and unpredictable surfaces on anteroposterior stability, which is likely due to impaired proprioceptive feedback and increased vestibular challenge [20]. This suggests that balance training and rehabilitation protocols should consider the complexity of surface conditions to effectively enhance postural control mechanisms.

Our findings reveal a distinction in COP velocity across different surface conditions, suggesting the nuanced ways in which the human body modulates its response to maintain stability [21]. The lower velocity on flat and incline conditions indicates a more measured and controlled postural adjustment strategy, likely facilitated by effective integration of sensory information [15,22]. In contrast, the results suggest that higher velocity on balance pads may indicate a less efficient balance maintenance strategy under heightened sensory uncertainty, as quicker and more reactive adjustments are required [23]. By comparing perturbation areas across surface conditions, we investigated the spatial dynamics of balance control. Smaller areas on flat and incline conditions suggest superior balance control by efficiently containing movement [24]. Conversely, larger areas observed on balance pads highlight the challenges faced by the human body in stabilizing its position, leading to broader movements as participants attempt to regain balance [6].

The findings of this study have practical implications for balance training and rehabilitation programs. The results suggest that incorporating diverse surface conditions in these programs can effectively challenge and enhance postural control strategies. Progressively increasing the difficulty of the surface conditions, starting from flat surfaces and moving towards inclined and compliant surfaces, can help individuals improve their ability to maintain balance in various real-life situations. Furthermore, the use of visual occlusion during training can provide additional challenges and help develop a more robust postural control system that is less reliant on visual feedback. Rehabilitation professionals can use these insights to design targeted interventions for individuals with balance impairments, focusing on the specific sensory and motor aspects that need improvement.

This study has several limitations that should be acknowledged. First, the generalizability of the findings may be limited due to the sample, which consisted of only healthy young adults, and the unequal sex distribution (20 males and 10 females). While the gender imbalance was a result of the voluntary participation and eligibility criteria, it may introduce potential bias. Future studies should aim for a balanced gender ratio to minimize this limitation. Second, as a cross-sectional study, it did not investigate the long-term adaptations of the postural control system to various surface conditions. Third, the study employed a limited set of surface conditions, and the impact of other types of surfaces remains unknown. Fourth, the study relied solely on COP displacement and velocity as indicators of postural stability, and integrating additional assessment methods could offer deeper insights. Finally, individual differences in sensory integration capabilities, postural control strategies, and factors such as sensory acuity, muscle strength, and anticipatory postural adjustments were not explored in this study. Future research should consider these factors to better understand the variability in balance performance under challenging surface conditions.

## 5. Conclusions

In conclusion, this study demonstrates that surface conditions significantly influence postural control strategies and stability. The key findings indicate that flat surfaces facilitate better postural stability compared to the incline ramp, balance pad, and balance pad on incline ramp conditions, as evidenced by lower COP displacements, velocities, and areas. These results suggest that the human postural control system is adaptable to various surface conditions but has limitations in maintaining stability in more challenging environments. The findings underscore the importance of considering surface conditions in balance assessment, training, and rehabilitation, implying that incorporating diverse surface types and perturbations in balance training programs may enhance an individual’s ability to adapt and maintain postural control in a wide range of environments. Furthermore, the study highlights the need for future research to investigate the long-term effects of surface-specific balance training on postural control and its potential implications for fall prevention and rehabilitation outcomes.

## Figures and Tables

**Table 1:** Demographic and anthropometric characteristics of the study participants.

Characteristics	Value
Number of Participants	30 (20 males, 10 females)
Age (years)	24.10 ± 1.84
Height (cm)	169.30 ± 6.55
Body mass (kg)	63.15 ± 8.82

**Table 2:** Comparison of the effects of different surface conditions (flat, incline ramp, balance pad, and balance pad on incline ramp) on COP displacement, velocity, and perturbation area during standing balance tasks with external perturbations.

Variables	Condition	Mean ± SD	F	*p*-Value	Effect Size
COP velocity (mm/s)	Flat	28.47 ± 4.50	89.177	<0.001 *	2.89
	Ramp	27.48 ± 4.38			
	Balance pad	49.76 ± 9.38			
	Balance pad on the ramp	49.03 ± 8.55			
COP area (mm^2^)	Flat	1104.07 ± 330.22	52.659	<0.001 *	2.15
	Ramp	1100.20 ± 500.79			
	Balance pad	2232.97 ± 778.91			
	Balance pad on the ramp	2202.30 ± 578.87			
COP ML (mm)	Flat	27.03 ± 7.23	38.272	<0.001 *	1.96
	Ramp	27.38 ± 8.43			
	Balance pad	42.10 ± 11.61			
	Balance pad on the ramp	44.03 ± 10.22			
COP AP (mm)	Flat	28.47 ± 4.50	89.177	<0.001 *	2.89
	Ramp	27.48 ± 4.38			
	Balance pad	49.76 ± 9.38			
	Balance pad on the ramp	49.03 ± 8.55			

The asterisk (*) indicates a statistically significant difference (*p* < 0.05) between the surface conditions for each variable.

## Data Availability

The data presented in this study are available on request from the corresponding author due to privacy and ethical restrictions related to participant confidentiality.
